# BMGE (Block Mapping and Gathering with Entropy): a new software for selection of phylogenetic informative regions from multiple sequence alignments

**DOI:** 10.1186/1471-2148-10-210

**Published:** 2010-07-13

**Authors:** Alexis Criscuolo, Simonetta Gribaldo

**Affiliations:** 1 Institut Pasteur, Unité de Biologie Moléculaire du Gène chez les Extrêmophiles, Département de Microbiologie, 25 rue du Dr Roux, 75015 Paris, France

## Abstract

**Background:**

The quality of multiple sequence alignments plays an important role in the accuracy of phylogenetic inference. It has been shown that removing ambiguously aligned regions, but also other sources of bias such as highly variable (saturated) characters, can improve the overall performance of many phylogenetic reconstruction methods. A current scientific trend is to build phylogenetic trees from a large number of sequence datasets (semi-)automatically extracted from numerous complete genomes. Because these approaches do not allow a precise manual curation of each dataset, there exists a real need for efficient bioinformatic tools dedicated to this alignment character trimming step.

**Results:**

Here is presented a new software, named BMGE (Block Mapping and Gathering with Entropy), that is designed to select regions in a multiple sequence alignment that are suited for phylogenetic inference. For each character, BMGE computes a score closely related to an entropy value. Calculation of these entropy-like scores is weighted with BLOSUM or PAM similarity matrices in order to distinguish among biologically expected and unexpected variability for each aligned character. Sets of contiguous characters with a score above a given threshold are considered as not suited for phylogenetic inference and then removed. Simulation analyses show that the character trimming performed by BMGE produces datasets leading to accurate trees, especially with alignments including distantly-related sequences. BMGE also implements trimming and recoding methods aimed at minimizing phylogeny reconstruction artefacts due to compositional heterogeneity.

**Conclusions:**

BMGE is able to perform biologically relevant trimming on a multiple alignment of DNA, codon or amino acid sequences. Java source code and executable are freely available at ftp://ftp.pasteur.fr/pub/GenSoft/projects/BMGE/.

## Background

Most phylogenetic inference approaches are based on an alignment of homologous sequences (e.g. DNA, RNA, amino acids). The alignment of sequences aims at highlighting the substitutions that have occurred during the evolutionary process from their common ancestral sequence. The quality of a multiple sequence alignment can have a strong impact on the accuracy of the inferred phylogenetic tree, whatever the inference criterion used [1-4]. In spite of constant improvements of the multiple sequence alignment heuristics [5,6], an alignment can contain regions (i.e. sets of contiguous characters, also often called blocks [7,8]) where homology is ambiguous. Moreover, too divergent regions (even when correctly aligned) may induce a mutational saturation effect, which is an important source of bias for many phylogenetic reconstruction methods. In order to minimize the bias introduced by these problematic regions, a frequent approach is to detect and remove them from the multiple sequence alignment prior to phylogenetic analysis (e.g. [9-13]). Indeed, it has been observed that the removal of such regions allows more accurate trees to be inferred [7,8,14-16].

A current trend consists in reconstructing phylogenetic trees by using a large number of datasets of aligned sequences from many complete genomes. Phylogenetic trees are then reconstructed from these datasets in many contexts, such as the construction of gene tree databases [13], the inference of species trees based on a core-gene set [17,18] or the estimation of amino acid substitution matrices [19]. These different phylogenetic explorations are often based on (semi-)automated processes (e.g. [15,18,20]), requiring a software solution for each step of these computer pipelines. Given the importance of dataset quality, the use of practical and accurate software dedicated to alignment trimming task has become a real need.

In this paper, we present a novel software, named BMGE (Block Mapping and Gathering with Entropy), that identifies regions inside multiple sequence alignments that are suited for phylogenetic inference. BMGE computes a score for each character (i.e. amino acid, nucleotide or codon column), mainly determined by the entropy induced by the proportion of character states. To estimate realistic scores that take into account biologically relevant substitution processes (e.g. transition rates more frequent than transversions for DNA sequences, highest probability of changes between amino acids with physicochemical similarities), BMGE weights the entropy estimation with standard substitution matrices (e.g. PAM or BLOSUM). Averaging score values across the characters of the multiple sequence alignment allows identifying conserved (i.e. with low entropy-like score values) and highly variable/uncertain regions (i.e. with large entropy-like score values [15,21]). By removing such high entropy regions, BMGE returns trimmed datasets that allow the reconstruction of more accurate phylogenetic trees than the initial alignment, as shown by simulation studies.

In addition, BMGE also provides simple solutions to alleviate systematic artefacts caused by compositional heterogeneity. Most probabilistic phylogenetic inference methods make the assumption (among other more or less axiomatic ones) that the studied sequences arose from a common ancestral sequence following a stationary evolutionary process, i.e. the marginal probabilities of the character states remained constant over all sequences (e.g. [11,22]). Consequently, when phylogenetic trees are inferred from sequences with heterogeneous composition of character states, the violation of the stationary assumption may cause systematic errors [19,23-25]. BMGE is therefore able to perform RY-coding from a DNA sequence alignment [25], and to convert amino acid sequences into their corresponding degenerated codons according to the universal genetic code. These two recoding strategies may prove useful to minimize some biases when dealing with datasets with known heterogeneous composition across sequences. As these two recoding approaches use only the standard one-letter nucleotide alphabet [26] (see Table 1), the resulting datasets can be given to all phylogeny inference programs, in contrast to alternative recoding techniques based on non-standard alphabet cardinality such as the "Dayhoff classes" 6-residue alphabet [27,28] (see also [29] for discussion on other recoding schemes). Moreover, the use of degenerated codons allow fast inference of trees, in particular with Maximum Likelihood (ML) methods which are faster with nucleotide sequences than with amino acid ones. BMGE also implements a novel stationary-based trimming method that allows compositionally heterogeneous characters to be identified and removed. To do so, BMGE uses the Stuart's *χ*^2 ^matched-pairs test of marginal symmetry [30] that allows assessing the null hypothesis that two sequences are compositionally homogeneous [31], and iteratively performs character removal/addition steps until the Stuart's test assesses that each pair of sequences presents homogeneous composition. As shown by computer simulations with heterogeneous GC-content DNA sequences, this stationary-based trimming leads to unbiased phylogenetic trees.

**Table 1:** Character state coding used by BMGE

Nucleotide	1-letter code		
Adenosine	A		
Guanine	G		
Cytosine	C		
Thymine	T		

**Degenerated nucleotide**	**1-letter code**	**Meaning**	

Methyl	M	A or C	
Purine	R	A or G	
Weak (3 H bonds)	W	A or T	
Strong (3 H bonds)	S	C or G	
Pyrimidine	Y	C or T	
Keto	K	G or T	
	B	not A	
	D	not C	
	H	not G	
	V	not T	
Any	N or X	one of the 4 nucleotides	

**Amino acid**	**1-letter code**		**Degenerated codon** **3-letter code**

Alanine	A		GCX
Arginine	R		MGX
Asparagine	N		AAY
Aspartic acid	D		GAY
Cysteine	C		TGY
Glutamine	Q		CAR
Glutamic acid	E		GAR
Glycine	G		GGX
Histidine	H		CAY
Isoleucine	I		ATH
Leucine	L		YTX
Lysine	K		AAR
Methionine	M		ATG
Phenylalaline	F		TTY
Proline	P		CCX
Serine	S		WSX
Threonine	T		ACX
Tryptophan	W		TGG
Thyrosine	Y		TAY
Valine	V		GTX
			

**Degenerated amino acid**	**1-letter code**	**Meaning**	**Degenerated codon ** **3-letter code**

Aspartate	B	N or D	RAY
Glutamate	Z	Q or E	SAR
Any	X	one of the 20 amino acids	XXX

## Implementation

### Input/output files and sequence coding conversions

The input file for BMGE is a multiple sequence alignment in FASTA (or PHYLIP sequential) format. The user must indicate whether the sequences are amino acids or DNA (with standard one-letter coding [26,32]; see Table 1). It is also possible to consider DNA sequences as codons, which allows the multiple sequence alignment to be handled with amino acid substitution matrices. Selected (and/or removed) regions are written in an output file in several formats (i.e. FASTA, PHYLIP sequential, NEXUS). HTML output is also available to display selected sites as well as graphical representation of both entropy values and gap proportions.

Several sequence conversion options are also available: from DNA or codons to RY-coding [25], and from codons to translated amino acids (according to the universal genetic code). BMGE also allows converting an amino acid alignment into a nucleotide alignment by considering the corresponding degenerated codons (see Table 1). In practice, given an amino acid and its set of corresponding synonymous codons (following the universal genetic code), the degenerated codon is simply obtained, for each of the three codon positions *p*, by considering the nucleotides corresponding to the set of possible codons at position *p*. For example, isoleucine (I) can be encoded by the three codons ATA, ATC and ATT; therefore, the degenerated codon corresponding to this set of codons is ATH, knowing that the degenerated nucleotide H (i.e. A, C or T; see Table 1) represents the possible nucleotides at the third threefold degenerate position.

### Entropy-based character trimming

Given a multiple sequence alignment of *m *character length, BMGE computes, for each character *c *= 1,2,...,*m *, the frequency *g*(*c*) of gaps, as well as the diagonal matrix **∏ **^(*c*) ^where each diagonal entry is the relative frequency of each of the *r *possible character states (*r = *4 or 20, for DNA or amino acid sequences, respectively). So, BMGE computes the value *h*(*c*) for each character *c*, which is closely related to the von Neumann entropy [33] and is given by the following formula [34]:

(1) $$h(c)=-\text{trace}[\mu \Pi^{\left(c\right)}Slog_{r}(\mu \Pi^{\left(c\right)}S)],$$

where **S **is a similarity matrix (see below for more details) and *μ *= [trace (**∏**^(*c*)^**S**) ]^-1 ^is a normalizing factor so that trace (*μ***∏**^(*c*) ^**S**) = 1. To compute formula (1), BMGE uses the simpler equation

(2) $$h(c)=-\sum_{s=1\ldots r}\lambda_{s}^{\left(c\right)}\log_{r}\lambda_{s}^{\left(c\right)},$$

where the $\lambda_{s}^{\left(c\right)}$ parameters are the eigenvalues of the matrix *μ***∏**^(*c*)^**S **estimated via the JAMA package [35]. It should be stressed that the entropy normalization condition $\sum_{s=1\ldots r}\lambda_{s}^{\left(c\right)}=1$ is verified with the normalizing factor *μ*.

Knowing that *h*(*c*) = 0 indicates that character *c *is constant and that *h*(*c*) is as close to 1 as character *c *is unexpectedly variable (see below), BMGE looks for conserved regions of the multiple sequence alignment by smoothing the different *h*(*c*) values by using a sliding window of length 2*w*+1 (with *w *= 1 by default). For each *c *= 1, 2,...,*m *, the smooth  value is estimated by the weighted average

(3) $$\tilde{h}(c)=\frac{\sum_{i=\text{start}}^{\text{end}}[1-g(i)]h(i)}{\sum_{i=\text{start}}^{\text{end}}[1-g(i)]},$$

with start = max(1;*c*-*w*) and end = min(*m*;*c*+*w*), in order to give more weight to characters with few gaps, i.e. *g*(*c*) ≅ 0. After this smoothing operation, BMGE defines as conserved those characters *c *that have  value lower than a fixed threshold (0.5 by default). A conserved region is then defined as a set of contiguous conserved characters.

Then, the multiple sequence alignment is partitioned into successive conserved (*C *) and variable (i.e. non-conserved; *V *) regions, these being either a single character or a set of contiguous ones. Let *V_i _*be the *i*^th ^non-conserved region. By definition, *V_i _*is flanked by the two conserved regions *C_i _*and *C*_*i*+1_. In order to distinguish variable regions due to ambiguous alignment from those due to natural variation, the average $\tilde{h}$ value is computed for the region *C*_*i *_∪ *V*_*i*_∪ *C*_*i*+1 _by formula (3) with parameters 'start' and 'end' set as the first character of *C*_*i *_and the last character of *C*_*i*+1_, respectively. The consecutive regions *C*_*i *_, *V*_*i *_, *C*_*i*+1 _with less than 30% of gaps and with $\tilde{h}$ value lower than the fixed 0.5 threshold are then merged into a unique conserved region. Finally, BMGE iteratively performs these merging operations until no more variable region *V*_*i *_can be merged with its two flanking *C*_*i *_and *C*_*i*+1 _ones.

### On the use of similarity matrix

If the similarity matrix **S **in formula (1) is the identity matrix **I***_r _*, then h(*c*) is closely related to the well-known Shannon entropy [36], given by formula (2) where each $\lambda_{s}^{\left(c\right)}=\Pi_{ss}^{\left(c\right)}$ is simply the proportion of the character state *s *for character *c*. If the character *c *is constant, then *h*(*c*) = 0. On the other hand, if the character *c *is highly variable, then each of the *r *character states is present with a relatively high proportion (e.g. $\Pi_{ss}^{\left(c\right)}\approx 1/r$), implying that *h(c) *is close to 1, its maximal value. Therefore, *h *allows the level of variability of a character to be quantified [21]. Unfortunately, using *h *with the identity matrix **I***_r _*(as suggested in [15]) suffers from biases. For example, when considering amino acid sequences, if a given character *c*_1 _is only made of the four residues I, L, M and V, each with 25% proportion, and a second character *c*_2 _is made of the four residues C, Q, W and Y, also with identical proportions, then formula (1) with matrix **I***_r _*returns *h*(*c*_1_) = *h*(*c*_2_) = -4× 0.25 log_20 _0.25 ≈ 0.462, and then indicates the same level of variability for both characters *c*_1 _and *c*_2_, while residues in character *c*_1 _are much more likely to be substituted than those in character *c*_2 _[27,37,38]. In contrast, using dedicated similarity matrices **S *≠ *I **allows relevant substitution processes to be taken into account. As suggested in [34], when using the Henikoff and Henikoff's [39] BLOSUM50 target frequency matrix in formula (1), one obtains *h*(*c*_1_) ≈ 0.300 and *h*(*c*_2_) ≈ 0.453. Therefore, the function *h *with appropriate similarity matrix **S **computes score values that allow distinguishing among expected (i.e. biologically relevant) and ambiguous (e.g. source of noise) variability.

For practical use with amino acid sequences, BMGE provides the complete range of BLOSUM target frequency matrices (i.e. BLOSUM30, 35, 40, ..., 95 from [40]; see [39] for more details) in order to estimate pertinent *h *values depending on the level of divergence between sequences. As shown in simulation results (see below), our entropy-based character trimming method performs better when using stringent matrices (e.g. BLOSUM95) for closely related sequences, and, reciprocally, when using more relaxed matrices (e.g. BLOSUM30) for distantly related sequences. By default, BMGE uses the popular BLOSUM62 matrix [41].

For DNA sequences, PAM matrices are first computed. BMGE uses a transition/transversion ratio *κ *(= 2.0 by default) to compute the PAM-1 4 × 4 matrix: the four diagonal elements are all 0.99, and the off diagonal elements are 0.01 *κ *(2 + *κ*)^-1 ^for transition and 0.01(2 + *κ*)^-1 ^for transversion (see [42] for more details about the PAM-1 calculation for DNA). Given a prefixed integer *η *(= 100 by default), BMGE then computes the PAM-*η *matrix (= PAM-1*^η ^*[27]), which is finally used by BMGE as similarity matrix **S **in formula (1). In a similar way as the previously described amino acid framework, our character trimming method is more accurate when using a stringent matrix (e.g. PAM-1) with closely related DNA sequences, and when using a relaxed matrix (e.g. PAM-250) with distantly related ones.

### Stationary-based character trimming

Given two aligned sequences (e.g. taken from the multiple sequence alignment), one can use a *r×r *divergence matrix **F **to represent the relative proportion of each of the possible character state pairs in the pairwise comparison of these two sequences (as schematized for DNA sequences by formula (19) in [11]). If these two sequences have similar character state composition, then, for each character state *s *= 1, 2,...,*r *, this sequence pair verifies the null hypothesis **F**_*s. *_= **F**_*.s *_, named the marginal homogeneity (e.g. [30,43]) or marginal symmetry (e.g. [31]). A not-too-low *p*-value returned by the Stuart's *χ*^2 ^test [30] on **F **allows the marginal homogeneity/symmetry to be assessed; then, one can assess that two homologous sequences arise from a nonstationary evolutionary process if the Stuart's test returns a *p*-value close to zero (e.g. < 0.1). This test being essentially based on numerical linear algebra operations (see e.g. [31] for more details about its computation from two sequences), BMGE implements it with, on the one hand, matrix operations available in the JAMA package [35], and, on the other hand, with fast and accurate numerical algorithms for estimating *χ*^2 ^cumulative distribution functions (adapted in Java from the C code sources available p. 216-219 in [44]).

If a multiple sequence alignment seems to be compositionally heterogeneous, BMGE implements a stationary-based character trimming in order to obtain a compositionally homogeneous alignment. Given a multiple alignment of *n *sequences, for each possible pair of distinct sequences *i*, *j *(i.e. 1 ≤ *i *<*j *≤ *n *), BMGE computes the Stuart's test *p*-value, denoted *p_ij_*. If there is at least one pair of sequences for which *p_ij _*< 0.1, then BMGE progressively removes the characters *c *ranked in function of their decreasing entropy-like *h*(*c*) values --as estimated by formula (1)-- until *p_ij _*> 0.1 for every pairs of sequences *i*, *j*. This first crude character removal approach leads to a set *C *of compositionally homogeneous characters. Then, BMGE aims at integrating the set *C *with some of the previously removed characters, in order to obtain a set of compositionally homogeneous characters of maximal size.

To do so, BMGE uses an iterative add-and-remove approach of characters. Defining *p_ij_*^(*c*) ^as the Stuart's test *p*-value for the sequence pair *i*, *j *after adding the character *c *inside the set *C*, BMGE computes the following score for each character *c *∉ *C *:

$$\sigma (c)=\sum_{1\leq i<j\leq n}\log(p_{ij}^{\left(c\right)}/p_{ij}).$$

If σ (*c*) > 0, then adding character *c *inside *C* leads to an increase for most of the *n(n-1)*/2 *p*-values; reciprocally, adding characters *c *with σ (*c*)< 0 leads to a (not wished) overall decrease of the *p*-values. BMGE then progressively removes the characters *c *∉ *C *from the initial multiple sequence alignment following the increasing order of their respective σ(*c*) value, i.e. from the smaller (negative) to the larger (positive) σ score value. After each character removal, every *p_ij _*are re-estimated. When all *p_ij _*> 0.1, BMGE stops removing characters from the initial alignment and considers the remaining (i.e. not removed) characters as the new set *C*. Finally, BMGE iteratively performs these add-and-remove operations until the set *C *of compositionally homogeneous characters cannot be increased further.

## Results and Discussion

In order to assess the utility of multiple alignment character trimming to infer more accurate phylogenetic trees and to compare the respective performances of BMGE (with different similarity matrices) with other available character trimming methods (i.e. Gblocks [7]; Noisy [14]; trimAl [16]), we carried out computer simulations and real case studies. Protocol and results are described below.

### Simulation results with entropy-based character trimming

The 200 first 40-taxon trees available in [45] were selected as model trees to generate artificial amino acid sequence datasets. On the one hand, in order to simulate phylogenetically informative characters, 10 clusters of 40 sequences each were generated using Seq-Gen [46] under the JTT model [47] from each of the 200 model trees. Sequence lengths for each of these 10 sequence clusters were randomly drawn from 30 to 70 amino acids. On the other hand, in order to simulate uninformative/variable characters, amino acid sequences were generated in the same way from a 40-taxon star tree (i.e. a tree with 40 leaves and a unique internal node).

Following these two steps, for each of the 200 initial model trees, we generated 20 clusters of 40 amino acid sequences of lengths 50 ± 20, ten containing informative phylogenetic signal, and ten containing uninformative/variable signal. Finally, from each of these 200 sets of 20 sequence clusters, 10 clusters were randomly drawn and concatenated, in order to produce a dataset composed of 200 clusters of 40 sequences of 500-amino acid length on average, each containing an equal mixture of phylogenetically informative and uninformative regions.

This simulation procedure to generate 200 clusters of sequences containing 50% (on average) of uninformative/variable characters was repeated three times, each with initial model tree branch lengths (see [45,48] for more details) multiplied by a divergence factor of 1, 2 and 3, respectively, in order to mimic from closely- to distantly-related sequence clusters. Each of these (3 × 200=)600 clusters of simulated amino acid sequences were aligned with MUSCLE [49,50]. The average lengths of these multiple sequence alignments are 513, 561, and 573 for the levels of divergence ×1, ×2, and ×3, respectively.

The software BMGE was applied on these multiple sequence alignments with three similarity matrices: BLOSUM30, BLOSUM62 and BLOSUM95. As a comparison, character trimming was also performed by using three available softwares.

Gblocks (0.91 b) was used with 'strict' and 'relaxed' parameter sets (see [7] for a precise description of these two parameter sets). However, the 'strict' conditions (default parameters in Gblocks) are indeed very stringent (e.g. no character was selected in ≈40% of the multiple sequence alignments with level of divergence ×3), and phylogenetic trees inferred from the remaining blocks (when these existed) were always less accurate than those inferred from the blocks returned by the 'relaxed' conditions (results not shown). Worse results than those obtained with the 'relaxed' conditions were also observed with alternative parameter sets (e.g. those described by [12]; results not shown). Then, results from Gblocks presented below are only those obtained with the 'relaxed' conditions.

The software Noisy was used with the --nogap options. Several other options were tested (especially the --cutoff one; see [14] for more details) but the Noisy default options allows better results to be observed with our simulated datasets.

The software trimAl (1.2rev59) allows three trimming methods (among numerous ones) to be used: 'gappyout', 'strictplus' and 'automated1'. Since the 'gappyout' method mainly focuses on highly gapped regions, this approach removed too few characters in our poorly gapped simulated datasets. Consequently, the 'gappyout' results are not shown since they are very close to those observed with the initial (i.e. non-trimmed) multiple sequence alignments.

**Figure 1:**
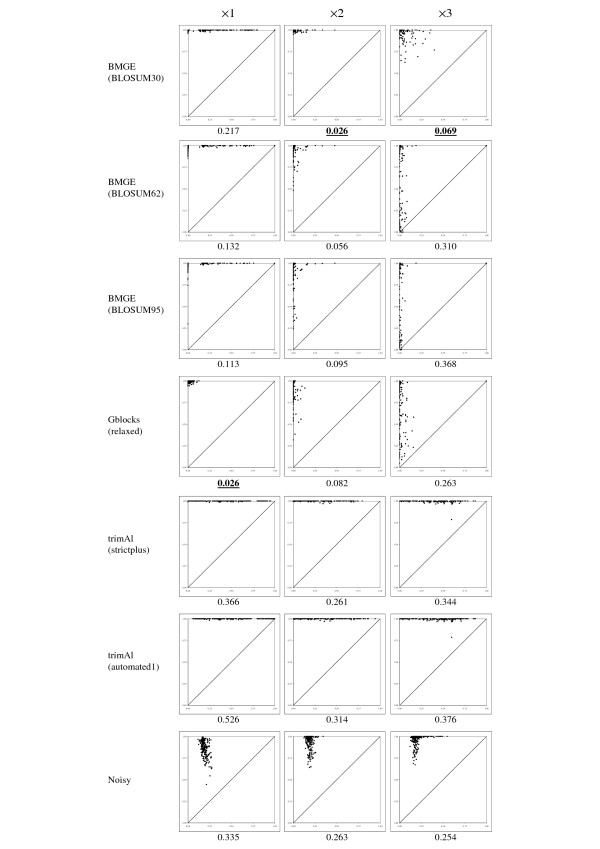
**ROC graphs plotting true (y-axis) and false (x-axis) positive rates for seven character trimming methods**. The best methods (i.e., that minimize both the number of true negative and false positive characters) are those with the corresponding cloud concentrated around the upper left point (0,1). Under each ROC graph, the average *L*_1 _distance between each point and the (0,1) point is given. For each level of divergence, the best (i.e. lower) distance is written in boldface characters. Average *L*_1 _distances that are not significantly different to this best value (as assessed by a sign test) are underscored.

As expected in regard to the sequence simulation protocol (see above), initial multiple sequence alignments returned by MUSCLE are composed of approximately 50% phylogenetically informative characters (i.e. those characters that are not generated by Seq-Gen from the star tree). For each trimming method, the character set of an initial multiple sequence alignment is then partitioned into four subsets: true positives and false negatives (i.e. phylogenetically informative characters that are selected or not by the trimming method, respectively), false positives (i.e. characters selected by the trimming method and that are not phylogenetically informative), and true negatives (i.e. characters that are not phylogenetically informative and are indeed not selected by the trimming method). Denoting *tp *and *tn *, the number of true positive and negative characters, respectively, and *fp * and *fn *, the number of false positive and negative characters, respectively, the true positive rate *tpr = tp/*(*tp *+ *fn*) and false positive rate *fpr = fp/*(*fp *+ *tn*) were computed. For each trimming method and each of the three levels of divergence (i.e. ×1, ×2, ×3), a ROC graph (e.g. [51-54]) depicting the plot of the true (y-axis) and false (x-axis) positive rates for each of the 200 datasets is represented in Figure 1.

All initial (i.e. non-trimmed) multiple sequence alignments (i.e. *tn = fn = *0) correspond to the (1,1) point (i.e. *fpr = fp/fp = tpr = tp/tp =*1); reciprocally, the removal of all characters (i.e. *tp = fp = *0) corresponds to the (0,0) point (i.e. *fpr = *0/*tn = tpr = *0/*fn *= 0). A cloud close to the (1,1) point in the ROC graph then indicates that the corresponding trimming method is liberal (i.e. it keeps too many uninformative/variable characters). Conversely, conservative trimming methods (i.e. that remove too many phylogenetically informative characters) correspond to clouds close to the (0,0) point. Ideally, the best method selects only those characters that are phylogenetically informative (i.e. *fn = fp = *0), and then corresponds to the (0,1) point inside the ROC graph (i.e. *fpr = *0/*tn = *0 and *tpr = tp/tp = *1). For each case in Figure 1, the *L*_1 _distance between each point and the (0,1) point (= 1-*tpr+fpr *) was computed, averaged and written under its ROC graph. For each of the three levels of divergence (i.e. ×1, ×2, ×3), a sign test [55-57] was performed to assess the statistical significance between the best average *L*_1 _distance measure (i.e. the lowest) and the other ones. For each level of divergence in Figure 1, an average *L*_1 _distance measure is considered as non-significantly different to the best one if the *p*-value returned by the sign test is > 5%.

Figure 1 shows that, in all cases, trimAl has always *tpr *≈ 1 but often *fpr *≫ 0, indicating that it is too liberal (i.e. *fp *≫ 0); moreover, *L*_1 _distances observed for trimAl are often among the worst, similarly to those observed for Noisy (except for the level of divergence ×3). We can also see from Figure 1 that Gblocks with relaxed conditions presents very good performance with closely related sequences (i.e. level of divergence ×1), but induces *tpr *«1 when the level of divergence increases (i.e. from ×1 to ×3), showing that it becomes too conservative (i.e. *fn *≫ 0). As expected, BMGE with stringent option (i.e. BLOSUM95) corresponds to points that are more concentrated around the y-axis as the level of sequence divergence increases, showing that the use of stringent similarity matrices such as ranging from BLOSUM80 to BLOSUM95 lead to conservative character trimming with distantly related sequences (e.g. level of divergence ×3). Reciprocally, the use of the similarity matrix BLOSUM30 leads to selecting too many characters (i.e. *fpr *≫ 0) with closely related sequences (i.e. level of divergence ×1). However, BMGE with BLOSUM30 allows minimizing both *fn *and *fp *when the level of divergence increases; indeed this last BMGE usage leads to the best average *L*_1 _distances for the levels of divergence ×2 and ×3.

### Simulation results on phylogenetic tree accuracy

For each of the three levels of divergence and from each multiple sequence alignment (i.e. the initial one as well as those outputted by BMGE, Gblocks, Noisy and trimAl), phylogenetic trees were inferred with several approaches. Maximum Likelihood (ML) trees were inferred by PhyML [48] with model JTT-Γ_4_. BioNJ [58] trees were also inferred by PhyML with JTT distances. Maximum Parsimony (MP) trees were inferred by TNT [59] with TBR branch swapping and parsimony ratchet [60]. Bayesian inference was not performed because of the huge running time required by this approach. Moreover, Bayesian trees are expected to be very close to ML trees when inferred from our simulated datasets [3].

For each of the three levels of divergence, topological accuracy of the ML, BioNJ and MP trees inferred from the initial and the trimmed multiple sequence alignments was measured by the quartet distance [61] between each inferred tree and its corresponding model tree. All these distance measures are reported in Tables 2, 3 and 4 for ML, BioNJ and MP trees, respectively, normalized in order to restrict these values to the interval [0,1], then averaged. For each of the three levels of divergence (i.e. ×1, ×2, ×3) and each of the three tree reconstruction methods (i.e. ML, BioNJ, MP), a sign test was performed to assess the statistical significance between the best average distance measure (i.e. the lowest) and the other ones. In each column of the Tables 2, 3 and 4, an entry is considered as non-significantly different to the best one if the *p*-value returned by the sign test is > 5%.

**Table 2:** Average quartet distances between the model trees and the ML trees inferred from the different trimmed multiple sequence alignments

		Level of divergence		
		
		×1	×2	×3
initial		0.0477	0.0457	0.0609
BMGE	(BLOSUM30)	0.0462	**0.0383**	**0.0427**
BMGE	(BLOSUM62)	**0.0444**	0.0384	0.0859
BMGE	(BLOSUM95)	0.0448	0.0404	0.0935
Gblocks	(relaxed)	0.0445	0.0414	0.0584
trimAl	(strictplus)	0.0462	0.0445	0.0539
trimAl	(automated1)	0.0457	0.0436	0.0519
Noisy		0.0621	0.0437	0.0430

For each column, the best (i.e. lower) distance is written in boldface characters. Average quartet distances that are not significantly different to this best value (as assessed by a sign test) are underscored.

**Table 3:** Average quartet distances between the model trees and the BioNJ trees inferred from the different trimmed multiple sequence alignments

		Level of divergence		
		
		×1	×2	×3
initial		0.1500	0.2110	0.2441
BMGE	(BLOSUM30)	0.0967	0.0669	**0.0861**
BMGE	(BLOSUM62)	0.0646	0.0668	0.1073
BMGE	(BLOSUM95)	0.0626	**0.0651**	0.1164
Gblocks	(relaxed)	**0.0569**	0.0668	0.0988
trimAl	(strictplus)	0.1027	0.1271	0.1798
trimAl	(automated1)	0.1055	0.1293	0.1820
Noisy		0.0768	0.0892	0.1141

For each column, the best (i.e. lower) distance is written in boldface characters. Average quartet distances that are not significantly different to this best value (as assessed by a sign test) are underscored.

**Table 4:** Average quartet distances between the model trees and the MP trees inferred from the different trimmed multiple sequence alignments

		Level of divergence		
		
		×1	×2	×3
initial		0.1858	0.1639	0.1587
BMGE	(BLOSUM30)	0.1189	**0.0601**	**0.0780**
BMGE	(BLOSUM62)	0.0949	0.0610	0.3011
BMGE	(BLOSUM95)	0.0884	0.0630	0.3194
Gblocks	(relaxed)	**0.0677**	0.0608	0.1026
trimAl	(strictplus)	0.1211	0.1085	0.1182
trimAl	(automated1)	0.1435	0.1142	0.1230
Noisy		0.1073	0.0965	0.0983

For each column, the best (i.e. lower) distance is written in boldface characters. Average quartet distances that are not significantly different to this best value (as assessed by a sign test) are underscored.

Variable/uncertain characters contained in the initial multiple sequence alignments cause strong artefacts in the resulting phylogenetic trees, especially for BioNJ and MP trees (see Tables 2, 3,and 4). When character trimming softwares are used, almost all reconstructed phylogenetic trees are closer to their model tree (Tables 2, 3, and 4), showing that character trimming is a useful step prior any phylogenetic inference. However, it should be stressed that the ML approach is very robust to the noise introduced by uninformative/variable characters in our simulated datasets, mainly thanks to the Γ parameter. Even if our datasets were generated with equal rates across characters (see above), estimation of a Γ parameter was included in ML inference because preliminary tries without Γ parameter led to less accurate trees, especially those inferred from Noisy and trimAl outputs (results not shown). The Γ parameter then helps to compensate part of the phylogenetic noise contained in our datasets. However, when considering distantly-related sequences (e.g. level of divergence ×3), the ML approach (as well as the BioNJ and MP ones) needs a preliminary trimming step to infer significantly accurate trees, in agreement with results from previous simulation-based studies (e.g. [8]).

In general, BMGE (when used with a BLOSUM substitution matrix adequate to the level of sequence divergence) allows reconstructing among the most accurate trees for each of the three levels of divergence and every tree reconstruction method used (Tables 2, 3 and 4). trimAl and Noisy infer the worst BioNJ and MP trees, whereas Gblocks with relaxed conditions produces good results as long as sequences are not too divergent. To the minor exception of Noisy with ML trees, BMGE trimming with the (less stringent) BLOSUM30 matrix allows reconstructing the significantly best trees with level of divergence ×3.

### Simulation results on phylogenetic tree branch support

In order to observe the impact of character trimming methods on branch supports inside phylogenetic trees, we have focused on two different approaches to estimate confidence values on the internal branches of a phylogenetic tree: the bootstrap-based support [62] with BioNJ trees, and the approximate likelihood ratio test (aLRT; [63]) as implemented by default in PhyML 3.0 [64].

**Figure 2:**
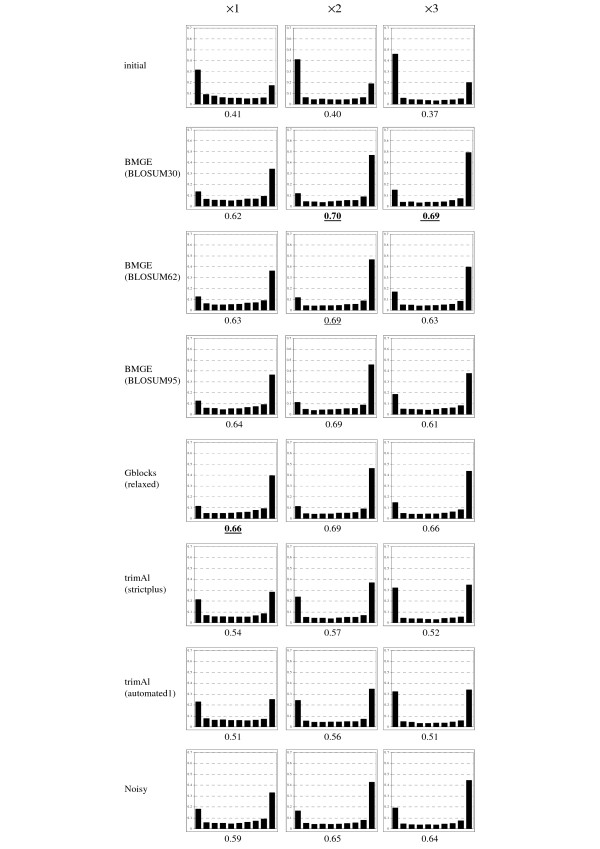
**Distributions of the BioNJ bootstrap-based confidence values on the true branches of model trees estimated from initial (non-trimmed) multiple sequence alignments and from alignments returned by seven character trimming methods**. Average confidence values are written under each corresponding histogram. For each level of divergence, the best (i.e. higher) average confidence value is written in boldface characters. Average confidence values associated to distributions that are not significantly different to this best distribution (as assessed by a *χ^2 ^*test) are underscored.

**Figure 3:**
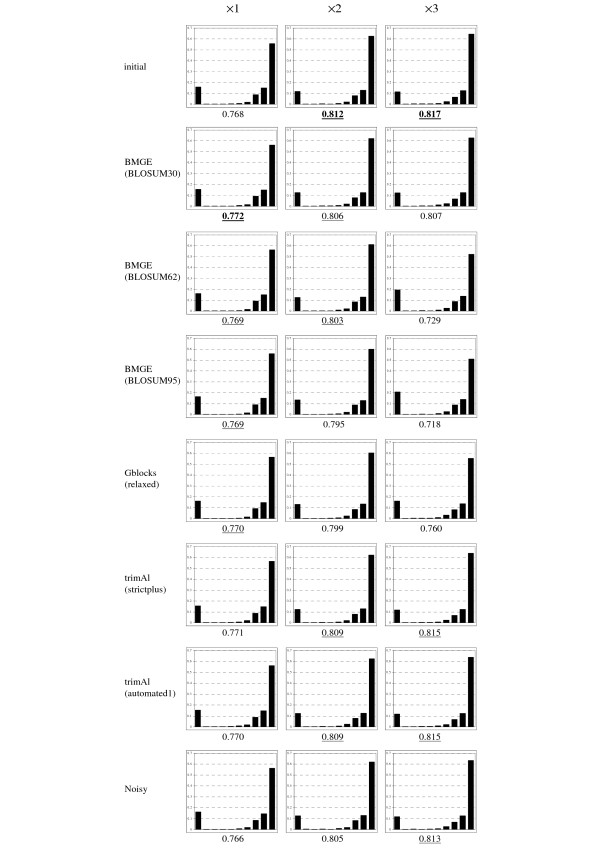
**Distributions of the aLRT confidence values on the true branches of model trees estimated from initial (non-trimmed) multiple sequence alignments and from alignments returned by seven character trimming methods**. See Figure 2.

For each of the three levels of divergence (i.e. ×1, ×2, ×3) and from each multiple sequence alignment (i.e. the initial as well as the seven outputted by BMGE, Gblocks, Noisy and trimAl; see above), 100 bootstrap-based replicates were generated, and 100 BioNJ trees were inferred from these multiple sequence alignment replicates. From these 100 BioNJ bootstrap-based trees, bootstrap proportions were assessed on the internal branches of the corresponding (true) model tree. For each character trimming method and each level of divergence, we computed the distribution of the so-obtained bootstrap-based confidence values, as well as its average value (Figure 2). Similarly, we also computed the distributions of aLRT branch support estimated on the (true) branches of the model trees from the different multiple sequence alignments, as well as their average values (Figure 3). For each way to estimate confidence values (i.e. BioNJ bootstrap-based and aLRT-based ones) and each level of divergence (i.e. ×1, ×2, ×3), a *χ*^2 ^test was performed to assess whether distributions are significantly different. In Figures 2 and 3, a distribution is considered as non-significantly different to the best one (i.e. those corresponding to the highest average confidence value) if the *p*-value returned by the *χ*^2 ^test is > 5%.

**Figure 4:**
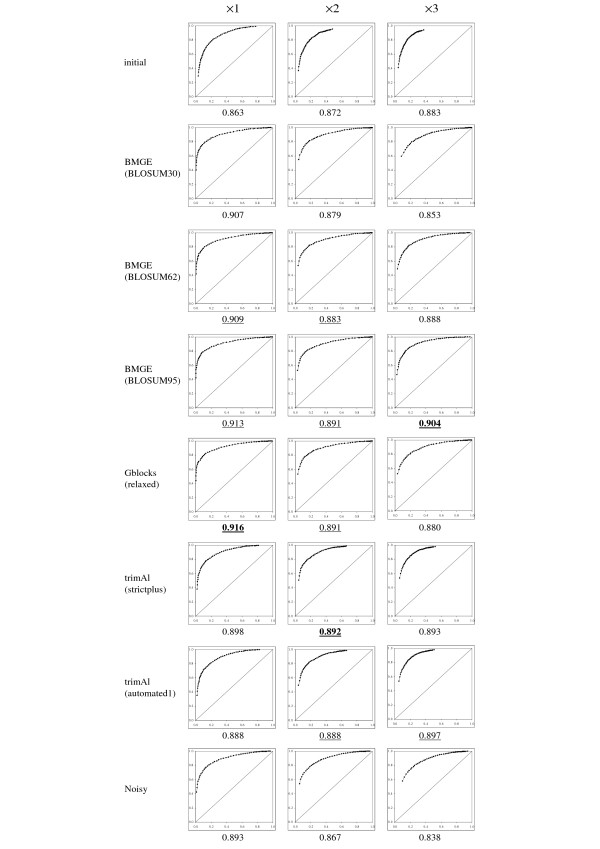
**ROC curves constructed by thresholding BioNJ bootstrap-based confidence values**. Best tree branch classifiers (i.e., that are able to associate higher confidence values to true branches than to false branches) are those that maximize the area under the ROC curve (AUC). For each simulation case, the AUC is given under the corresponding ROC space representation. For each column, the best (i.e. higher) AUC is written in boldface characters. AUCs that are not significantly different to this best value (as assessed by a Z test) are underscored.

**Figure 5:**
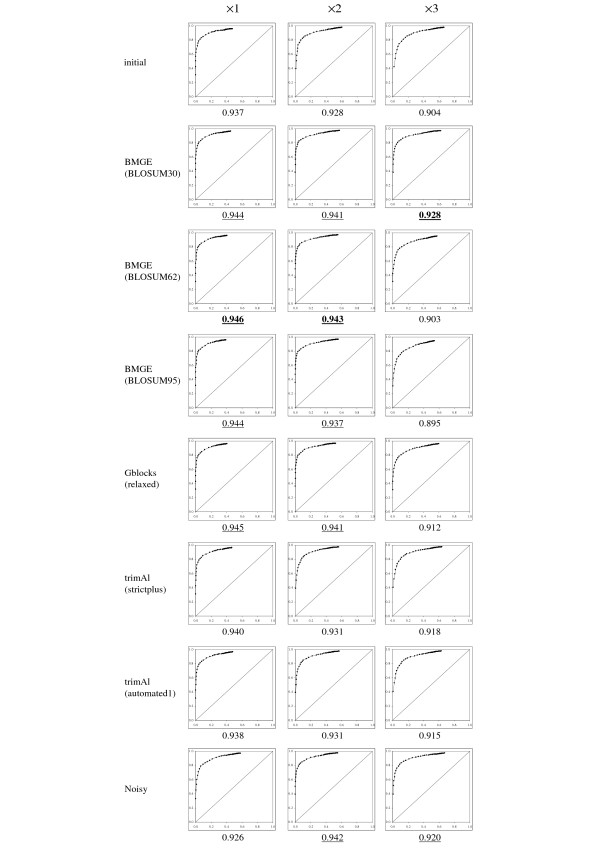
**ROC curves constructed by thresholding aLRT-based confidence values**. See Figure 4.

For each of the three levels of divergence and from each multiple sequence alignment, BioNJ-based bootstrap proportions were also estimated on the branches of the phylogenetic trees inferred by BioNJ (i.e. those used to compute quartet distances in Table 3). A bootstrap-based confidence value is then expected to be high when the corresponding branch is true (i.e. present in the model tree), and is expected to be low for false branches. Given a threshold *τ*, all branches of an inferred tree can be partitioned into four subsets: true positives or false negatives (i.e. true branches with confidence values ≥ *τ *or <*τ*, respectively), false positives or true negatives (i.e. false branches with confidence values ≥ *τ *or <*τ*, respectively). As a consequence, there exists a point in the ROC space (see above) that is associated to a threshold value *τ*, and plotting these points for a large number of possible threshold values *τ *results in a so-called ROC curve (see [52,54] for more details). ROC curves obtained by varying τ from 0 to 1 with 0.02-increment are displayed in Figure 4, where up-right head of each ROC curve corresponds to lowest *τ *values, whereas down-left tail corresponds to highest *τ *values. Figure 5 represents ROC curves built in the same way from aLRT-based confidence values on the branches of the inferred ML trees (i.e. those used to compute quartet distances in Table 2). Figure 4 and 5 also display the area under each ROC curve (AUC) that corresponds to the probability that a confidence value will be higher for a true branch than for a false branch (see [65,54] for more details). For each of the three levels of divergence, a Z test (as described in [66]) was performed to assess the statistical significance between the best AUC (i.e. the highest) and the other ones. In Figures 4 and 5, an AUC is considered as non-significantly different to the best one if the *p*-value returned by the Z test is > 5%.

Broadly, Figure 2 shows that the estimate of BioNJ bootstrap proportions from the initial (non-trimmed) multiple sequence alignments leads to very biased values, with a proportion of true branches with bootstrap-based confidence values ≤ 0.1 varying from 31% to 47%, and those > 0.9 varying from 18% to 20%. Figure 2 also shows that the highest average bootstrap-based confidence values are obtained from the character-trimming methods that best optimize both *tpr *and *fpr *criteria (see above and Figure 1). The same conclusions hold for aLRT with level of divergence ×1 (Figure 3), with (significantly) best average aLRT values observed with BMGE and Gblocks. Surprisingly, for level of divergence ×3, the best average aLRT values are obtained from the characters selected by the most liberal character trimming methods, i.e. initial alignments and trimAl (strictplus and automated1).

AUC values in Figures 4 and 5 show that initial (non-trimmed) multiple sequence alignments lead to more incorrect confidence values than those estimated from characters selected by trimming methods. Interestingly, Figures 4 and 5 show that aLRT-based ROC curves induce highest AUC values on average that bootstrap-based ones (with the slight exception of BMGE with BLOSUM95 for level of divergence ×3). This shows that aLRT-based confidence values are more able to discreminate true and false branches than BioNJ bootstrap-based ones. ROC curve shapes also show that trimming methods lead to more precise confidence values. Indeed, in Figure 4, the down-left tail point of each ROC curve (i.e. corresponding to threshold *τ *= 0.98) always has highest *tpr *(y-axis) for trimmed alignments than for initial ones; this shows that a larger proportion of true branches with BioNJ bootstrap-based confidence values >0.98 is observed with trimmed alignments than with initial alignments. For initial multiple sequence alignments and for level of divergence ×2 and ×3 in Figure 4, the up-right head points of these two ROC curves (i.e. each corresponding to threshold *τ *= 0.02) have *tpr* < 1; this means that there exists some true branches with BioNJ bootstrap-based confidence value < 0.02. Notably, this is not the case when using character trimming methods. These two tendencies on *tpr *values for both extremities of the ROC curves are in agreement with the distributions in Figure 2. Nevertheless, when observing the *fpr *ranges (x-axis) of the ROC curves in Figure 4, trimmed multiple sequence alignments all induce larger *fpr *values than initial ones; this shows that when using character trimming methods instead of initial multiple sequence alignments, there is an increase in the proportion of false branches in BioNJ trees with high confidence value (e.g. down-left tail of ROC curves corresponding to *τ *= 0.98) and a decrease of the proportion of false branches with low confidence values (e.g. up-right tail of ROC curves corresponding to *τ *= 0.02). This last tendency is clearly obvious with level of divergence ×3 (see Figure 4). However, shapes of ROC curves constructed by thresholding aLRT-based confidence values on ML trees do not seems to be strongly modified by trimming methods (see Figure 5). More precisely, for levels of divergence ×1 and ×2, Gblocks and BMGE (with BLOSUM62) present always among the best results. For level of divergence ×3, Noisy and BMGE with BLOSUM30 present the significantly best AUC values for aLRT-based confidence values.

To sum up, these simulation results show that, by selecting among variable characters those with biologically-relevant expected variability thanks to the use of similarity matrices, BMGE often presents results that are among the (significantly) best (e.g. phylogenetic accuracy, better confidence values for true branches) and leads to a less biased phylogenetic signal.

### Entropy-based character trimming in a phylogenomics context

In order to illustrate the benefit of using character trimming approaches, we have re-analysed the multi-gene dataset used by Castresana (2000) to describe the usefulness of Gblocks [7] in selecting suited characters. This amino-acid dataset is composed by ten genes (i.e. three subsunits of cytochrome *c *oxidase CO1-CO3, one subunit of cytochrome *c*-ubiquinol oxidoreductase CYTb, and six subunits of NADH desydrogenase ND1-ND5 and ND4L) gathered from the complete mitochondrial genome of 16 eukaryotes (11 unikonts, 4 archaeplastida, 1 excavate), and from *Paracoccus denitrificans*, an α-proteobacterium used as outgroup (see [7] for more details).

Protein sequences were aligned with MUSCLE, and each of the 10 multiple sequence alignments were trimmed by BMGE, Gblocks, trimAl and Noisy with the same parameters used in the previous simulations. For each of these seven character trimming approaches as well as the initial (non-trimmed) multiple sequence alignment, the ten so-obtained character matrices were concatenated into a single character supermatrix with the software Concatenate [67]. Table 5 shows the different number of characters of these eight supermatrices. ML trees were inferred with PhyML from each character supermatrix with the mtREV model of amino acid substitution [68]. Parameters defining the shape of the Γ distribution (8 categories) and the proportion of invariable characters were both left as free. The different log-likelihood (log-*lk*) estimated for each inferred ML trees were normalized by the total number of characters for better comparison and are shown in Table 5. ML bootstrap-based (100 replicates) and aLRT confidence values were assessed on the branches of the different inferred trees.

**Table 5:** Lengths of the different character supermatrices, and average log-likelihood per character of the corresponding ML trees.

		Number of characters	log-*lk *per character
initial		4,530	-21.07
BMGE	(BLOSUM30)	2,995	-23.42
BMGE	(BLOSUM62)	2,535	-21.01
BMGE	(BLOSUM95)	2,343	-19.94
Gblocks	(relaxed)	2,908	-23.00
trimAl	(strictplus)	2,881	-22.46
trimAl	(automated1)	3,104	-23.46
Noisy		2,513	-18.55

**Figure 6:**
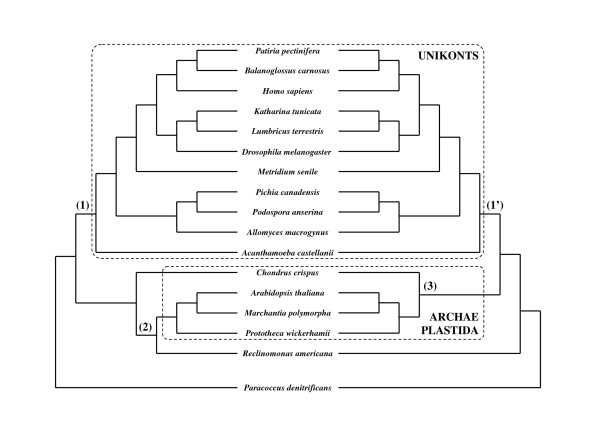
**Phylogenetic trees obtained from a non-trimmed character supermatrix (left) and from the concatenation of the multiple sequence alignments trimmed by BMGE with BLOSUM95 (right)**. These ML trees were inferred by PhyML with the model mtREV+Γ_8_+I. Note that the left topology was also inferred from character supermatrices built by concatenating multiple sequence alignments trimmed by BMGE (BLOSUM30 and BLOSUM62), Gblocks (relaxed), trimAl (strictplus and automated1), and Noisy. Bootstap-based and aLRT-based confidence values at nodes (1), (1'), (2) and (3) are given in Table 6.

It should be stressed that our initial character supermatrix (i.e. 4,530 characters; see Table 5) is slightly larger than the number of characters inside the original character supermatrix in [7] (i.e. 4,453); this is due to the use of different multiple sequence alignment softwares. It should also be stressed that the log-*lk *per character estimated from our initial character supermatrix (i.e. -21.07; see Table 5) is higher than those provided in [7] (i.e. -22.71; see Table 4, page 546 in [7]); this can be explained by the different number of characters but also by our use of the amino acid model mtREV+Γ_8_+I instead of just mtREV in [7]. However, the ML tree inferred from our initial character supermatrix has the same topology (left-hand tree in Figure 6) as the ML tree inferred by Castresana (2000; see Figure 5A, page 545 in [7]). The use of BMGE (with default BLOSUM62 and liberal BLOSUM30 matrices), Gblocks (relaxed), trimAl (strictplus and automated1) and Noisy lead to the same phylogenetic tree (left-hand tree in Figure 6). However, BMGE with the stringent BLOSUM95 similarity matrix leads to a different ML phylogenetic tree (right-hand tree in Figure 6). These two trees agree on the monophyly of Unikonts (nodes 1 and 1' in Figure 6), but differ in the placement of the jacobid *Reclinomonas americana*, which is inside the Archaeplastida subtree in the left-hand tree of Figure 6. Knowing that Archaeplastida and Unikonts are each likely monophyletic, and that jacobids are phylogenetically distinct from Archaeplastida (e.g. [69-72]), the BMGE (with BLOSUM95) tree at the right-hand side in Figure 6 seems more accurate. Moreover, BLOSUM95-based character trimming in BMGE gives among the best confidence values for the monophyly of Unikonts whereas Gblocks and trimAl both weakly support this subtree (see confidence values for nodes 1 and 1' in Table 6). The monophyly of Archaeplastida is weakly supported by all approaches, suggesting that this dataset does not induces sufficient phylogenetic signal for this particular node. However, BMGE with BLOSUM95 seems to provide a character trimming that leads to both accurate phylogenetic tree and confidence values, probably due to its ability to best minimize the number of false positive characters (see above and Figure 1).

**Table 6:** ML bootstrap-based (boot.) and aLRT-based confidence values derived from different character supermatrices.

		(1)		(1')	(2)		(3)	
					
		**boot**.	aLRT	aLRT	**boot**.	aLRT	**boot**.	aLRT
initial		0.60	0.389	**1.000**	0.92	0.919	0.07	0.000
BMGE	(BLOSUM30)	0.65	0.673	0.999	0.86	0.864	0.06	0.000
BMGE	(BLOSUM62)	0.78	0.725	**1.000**	0.42	0.296	0.29	0.000
BMGE	(BLOSUM95)	**0.83**	0.826	0.999	0.21	0.000	**0.46**	**0.711**
Gblocks	(relaxed)	0.64	0.311	0.999	0.80	0.895	0.10	0.000
trimAl	(strictplus)	0.59	0.379	0.999	0.91	**0.973**	0.03	0.000
trimAl	(automated1)	0.74	0.686	**1.000**	**0.93**	0.955	0.05	0.000
Noisy		0.81	**0.936**	**1.000**	0.77	0.776	0.22	0.000

Nodes (1), (1'), (2) and (3) are indicated on the phylogenetic trees in Figure 6. Nodes (1) and (1') correspond to the monophyly of Unikonts. Paraphyly and monophyly of Archaeplastida correspond to nodes (2) and (3), respectively. For each column, the best (i.e. higher) confidence value is written in boldface characters.

This re-analysis of a known phylogenomic dataset shows that it is quite difficult to choose an appropriate similarity matrix with BMGE, knowing that, in the one hand, these mitochondrial amino acid sequences are distantly related (see [7]), and that, in the other hand, a less unrealistic tree is inferred when BMGE is used with the stringent BLOSUM95 similarity matrix. To deal with amino acid sequences and BLOSUM matrices, a trivial approach would be to use the sequence clustering rule defined by Henikoff and Henikoff [39]: to compute the BLOSUM*η *similarity matrix, they grouped amino acid sequences into clusters in such a way that each sequence in any cluster has at least *η*% identity to at least one other sequence in this cluster. Given an alignment with *n *amino acid sequences, a systematic procedure to choose the BLOSUM *η *similarity matrix is then to apply the following formula:

(4) $$\eta =\underset{{}_{i=1,2,\ldots ,n}}{\min}\;\underset{{}_{j=1,2,\ldots ,i-1,i+1,\ldots ,n}}{\max}\{\%\;\;\text{identity}\;\text{between}\;\text{sequences}\;i\;and\;j\}$$

Unfortunately, this formula underestimates the value of *η* that best minimizes both the number of false positive and negative characters in our simulated datasets, e.g. *η*, as estimated by (4), varies from 16 to 71 with an average of 33 with level of divergence ×1, and varies from 10 to 62 with an average of 22 with level of divergence ×3, whereas simulation shows that best results are obtained with *η *= 95 and 30 for levels of divergence ×1 and ×3, respectively. Indeed, formula (4) on the Castresana (2000) phylogenomic dataset gives *η *values varying from 34 (ND3) to 64 (CO1), which shows that the amino acid sequences constituting these ten alignments are not closely related, whereas we have shown that the conservative BLOSUM95 similarity matrix is best adapted than BLOSUM62 and BLOSUM30 ones. Moreover, when estimated by (4) from the eight considered character supermatrices, *η *varies from 52 (concatenated initial multiple sequence alignments) to 60 (concatenated alignments trimmed by Noisy). A similar underestimation of *η *was also observed by averaging the *n *max values in formula (4), or by considering only characters with no gaps. Finally, even if formula (4) or related could give a suited approximate of the *η *value by using the building rules of BLOSUM matrices, it will be even more difficult to provide a similar formula for the PAM-*η *similarity matrices when dealing with DNA sequences.

Therefore, as maintained by Ewens and Grant (2005) for BLAST queries, we believe that "*one often has prior knowledge about the evolutionary distance between the sequences of interest that helps one choose which BLOSUM matrix to use*" (p. 244 in [73]). In practice, when inferring a particular gene tree, our opinion is to carefully examine the original multiple sequence alignment and those obtained by BMGE trimming with several similarity matrices (i.e. BLOSUM or PAM). On the contrary, when building a phylogenomic dataset, we believe, as Talavera and Castresana (2007), that "*there is enough information from the concatenation of several genes*" and then that "*stringent conditions tend to give rise to the best phylogenetic trees*" (p. 575 in [7]): the use of BMGE with stringent similarity matrices (e.g. BLOSUM95 or PAM-1) can strongly increase the number of false negatives (i.e. too many characters suited for phylogenetic inference are removed; see Figure 1) but systematic biases due to this conservative approach are often compensated by a sufficiently large number of genes used. Finally and in every case, we think that it is more relevant to deal with well-defined similarity matrices in order to choose among stringent to relaxed character trimming as in BMGE, rather than having to set several (and subjective) numerical parameters.

### Simulation results with stationary-based character trimming

**Figure 7:**
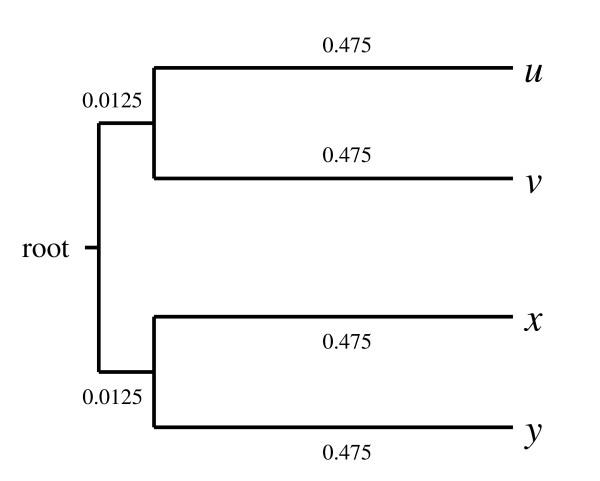
**Phylogenetic tree used to simulate the non-stationary evolution of a DNA sequence**. This tree and the different branch lengths are closely related to [24].

We illustrate the impact of compositional heterogeneity and stationary-based character trimming on the accuracy of phylogenetic tree inference with a simple computer simulation. Given the quartet tree in Figure 7, the evolution of a DNA sequence of length 10,000 with 25%-proportion of each nucleotide was simulated by Seq-Gen from the root to the four leaves *u*, *v*, *x *and *y*. To infer the evolution of this DNA sequence, the evolutionary model F81 [74] was chosen. On the one hand, Seq-Gen was used with equal relative character state frequencies to generate a compositionally homogeneous region (i.e. from 100% to 50% of the total length, with 10% increments). On the other hand, to generate a compositionally heterogeneous region (i.e. from 0% to 50% of the total length, respectively), DNA evolution was simulated with 80% GC-content for the external branches corresponding to the taxa *u *and *x*, and 20% GC-content for the two other external branches (i.e. taxa *v *and *y*). For each proportion of characters with heterogeneous composition (i.e. 0%, 10%, ..., 50% of the length of the alignment), 500 four-sequence alignments were generated following this procedure.

**Figure 8:**
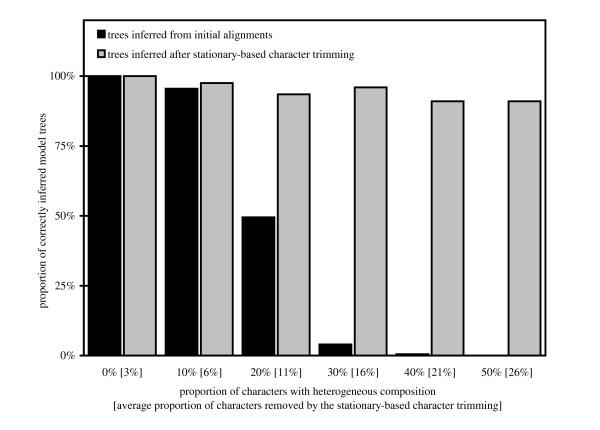
**Frequency of recovered model tree in function of the proportion of regions with heterogeneous composition inside an alignment of four DNA sequences**.

ML trees were inferred with PhyML (model F81) from all these simulated alignments. Stationary-based character trimming was applied with BMGE, and ML trees were inferred from the resulting compositionally homogeneous alignments. For each of the five proportions (i.e. 0%, 10%, ..., 50%) of unequal GC-content characters, the number of times that the model tree (Figure 1) was correctly inferred from both initial and trimmed alignments is graphically represented in Figure 8. The average proportions of characters removed by the stationary-based trimming are also reported. As expected [24], the model tree is often recovered with the initial alignments when these contain no or a small proportion of compositionally heterogeneous characters (e.g. < 20% of the total length of the initial alignment; see Figure 8), whereas the model tree is much less or not recovered when there is a high overall GC-content in taxa *u *and *x*, causing a biased attraction between them [25]. Thanks to the stationary-based character trimming, BMGE detects and removes regions that are compositionally heterogeneous; then, as shown in Figure 8, the model tree is very often recovered (e.g. the model tree is correctly inferred from more than 90% of the trimmed alignments), even when the proportion of unequal GC-content characters across sequences is high (e.g. > 30% of the total length of the initial alignment).

There exist many alternative statistical solutions to compare the character state composition of two (or more) sequences. Each of these has its own strengths and weaknesses (see [24] for instructive survey and discussion). However, matched-pairs tests (such as Stuart's test [30]) are comparatively efficient, particularly because of their ability to consider aligned sequences on a site-by-site basis [24]. Albeit the stationary-based character trimming may be extended by the use of overall tests for marginal symmetry (i.e. assessing the compositional homogeneity in the complete multiple sequence alignment [75,31]), we think that using pairwise *p*-values allows more precise sorting of the characters *c *according to their *σ*(*c*) score value (see above). Moreover, such overall tests are more time consuming. It should also be stressed that the Bowker's [76] test (i.e. another matched-pairs test assessing the complete symmetry inside **F**) was tried instead of the Stuart's test in the stationary-based trimming, but it led to worse results in the simulation analysis (not shown). Finally, as stressed in [43], Stuart's test is based on ordinary *χ^2 ^*approximation and is not appropriate for small samples, particularly when the number of categories (i.e. the row number in **F**) is not small. It is then strongly recommended to use the stationary-based character trimming on a large number of characters (e.g. ≥ 1, 000, such as in supermatrices of characters), especially when dealing with amino acid sequences.

### Real case study with stationary-based character trimming

In order to illustrate the performance of the stationary-based character trimming, we applied it on the multi-gene dataset described in [77] (available at [78]), which is known to suffer from a GC-content bias. This phylogenomic dataset was built by concatenating 106 alignments of DNA sequences gathered from 7 *Saccharomyces *species and *Candida albicans *as outgroup (see [77] for more details). The so-obtained supermatrix contains 127,026 nucleotide characters.

**Figure 9:**
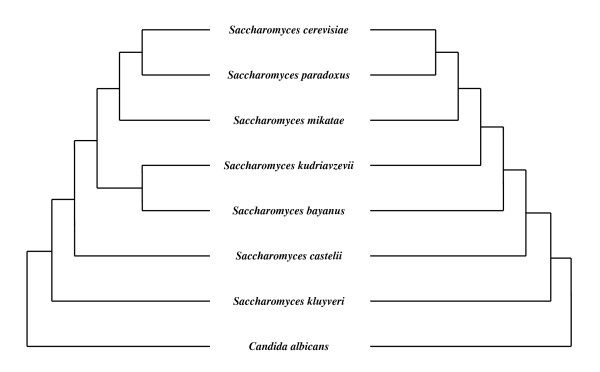
**Phylogenetic trees inferred before (left) and after (right) stationary-based character trimming**. Both trees are inferred by minimizing the Minimum Evolution criterion with GTR and LogDet distance estimations. ME bootstrap-based confidence values at branches are all 100%.

By using on this character supermatrix the same methods and software as Phillips, Delsuc and Penny (2004; see [25] for more details), we retrieved the same phylogenetic tree (left-hand tree in Figure 9) by minimizing the Minimum Evolution (ME) criterion with both GTR [79-81] and LogDet [82-84] distance estimates. We also retrieved the same ME bootstrap-based confidence values as in [25], i.e. 100% bootstrap proportion at all branches. Nevertheless, as shown by numerous phylogenetic analyses of the same dataset (e.g. [77,25,85-89]), this tree is incorrect: the real evolutionary history of these 8 yeast taxa is in fact the right-hand tree in Figure 9, with no monophyletic relationship between *S. kudriavzevii *and *S. bayanus*. It was shown in [25] that this systematic bias is due to a GC-content compositional heterogeneity across sequences that is sufficiently important to mislead the ME criterion.

A subset of 114,105 characters was selected by stationary-based character trimming implemented in BMGE. These so-selected characters were used to infer ME trees following the same methods as described previously. As expected, the right-hand tree in Figure 9 was inferred from both GTR and LogDet distance estimates with 100% bootstrap-based confidence value at each branch. More precisely, pairwise Stuart's test *p*-values are all ≈ 0 in the initial character supermatrix (with the slight exception of the two sequence pairs *S. cerevisiae - S. paradoxus *and *S. cerevisiae - S. mikatae *with *p*-values of 0.0146 and 0.0868, respectively). After the stationary-based trimming performed by BMGE, all *Saccharomyces *sequences induce pairwise Stuart's test *p*-values > 0.85, the lowest *p*-values being induced by the outgroup species (i.e. varying from 0.1000 to 0.3675 for the sequence pairs *C. albicans - S. kluyveri *and *C. albicans - S. castellii*, respectively). This shows that by removing **≈**10% characters, the stationary-based trimming is able to select a subset of compositionally homogeneous characters (as assessed by the pairwise Stuart's tests) that leads to unbiased ME phylogenetic inference.

## Conclusions

There exists a real need for accurate bioinformatic tools to extract at best the phylogenetic information contained in the ever-growing amount of available genomic sequence data. BMGE allows accurate character trimming of multiple alignments of DNA, codon, or amino acid sequences based on entropy-like scores weighted with BLOSUM or PAM matrices. Thus, BMGE is able to identify and extract unambiguously aligned blocks of characters that contain biologically expected variability and are therefore suitable for phylogenetic analysis. Simulation studies show that the trimmed datasets returned by BMGE lead to inference of accurate trees, in particular when in presence of multiple alignments including distantly-related sequences. BMGE also allows a number of useful recoding and trimming aimed at minimizing compositional heterogeneity in the alignment dataset and therefore the risk of phylogenetic artefacts.

In conclusion, BMGE is an accurate tool that can have several applications in phylogenomics analyses. The software BMGE is freely available (see below), and can also be used online through the Mobyle Web Portal [90] at http://mobyle.pasteur.fr/cgi-bin/portal.py.

## Availability and Requirements

**• Project name**: Block Mapping and Gathering with Entropy (BMGE)

**• Project home page**: ftp://ftp.pasteur.fr/pub/GenSoft/projects/BMGE/

**• Operating systems**: Platform independent

**• Programming language**: Java

**• Other requirements**: Java 1.6 or higher

**• License**: GNU General Public License (version 2)

**• Any restrictions to use by non-academics**: None

## Authors' contributions

AC initiated the project, designed and implemented the different methods inside the software, performed the computer simulations, and wrote the manuscript. SG supervised the project, and participated in designing the entropy-based character trimming method and in writing the manuscript. All authors read and approved the final manuscript.

## References

[B1] Lake JA The order of sequence alignment can bias the selection of tree topology Mol Biol Evol 1991 8 378 385 2072863 10.1093/oxfordjournals.molbev.a040654

[B2] Morrison DA Ellis JT Effects of nucleotide sequence alignment on phylogeny estimation: a case study on 18 S rDNAs of apicomplexa Mol Biol Evol 1997 14 428 441 9100373 10.1093/oxfordjournals.molbev.a025779

[B3] Ogden TH Rosenberg MS Multiple sequence alignment accuracy and phylogenetic inference Syst Biol 2006 55 314 328 10.1080/10635150500541730 16611602

[B4] Wang L-S Leebens-Mack J Wall PK Beckmann K dePamphilis CW Warnow T The impact of multiple protein sequence alignment on phylogenetic estimation IEEE/ACM Trans Comput Biol Bioinf 2009 in press 10.1109/TCBB.2009.68 21566256

[B5] Edgar RC Batzoglou S Multiple sequence alignment Curr Opin Struct Biol 2006 16 368 373 10.1016/j.sbi.2006.04.004 16679011

[B6] Notredame C Recent evolutions of multiple sequence alignment algorithms PLoS Comput Biol 2007 3 e123 10.1371/journal.pcbi.0030123 17784778 PMC1963500

[B7] Castresana J Selection of conserved blocks from multiple alignments for their use in phylogenetic analysis Mol Biol Evol 2000 17 540 552 10742046 10.1093/oxfordjournals.molbev.a026334

[B8] Talavera G Castresana J Improvement of phylogenies after removing divergent and ambiguously aligned blocks from protein sequence alignments Syst Biol 2007 56 564 577 10.1080/10635150701472164 17654362

[B9] Olsen GJ Woese CR Ribosomal RNA: a key to phylogeny FASEB J 1993 7 113 123 8422957 10.1096/fasebj.7.1.8422957

[B10] Rodrigo AG Bergquist PR Bergquist PL Inadequate support for an evolutionary link between the Metazoa and the Fungi Syst Biol 1994 43 578 584

[B11] Swofford DL Olsen GJ Waddell PJ Hillis DM Hillis DM, Moritz C, Mable BK Phylogenetic inference Molecular Systematics 1996 Sunderland: Sinauer Associates 407 514

[B12] Rodríguez-Ezpeleta N Brinkmann H Burey SC Roure B Burger G Löffelhardt W Philippe H Lang BF Monophyly of primary photosynthetic eukaryotes: green plants, red algae, and glaucophytes Curr Biol 2005 15 1325 1330 10.1016/j.cub.2005.06.040 16051178

[B13] Huerta-Cepas J Bueno A Dopazo J Gabaldón T PhylomeDB: a database for genome-wide collections of gene phylogenies Nucleic Acids Res 2008 36 D491 D496 10.1093/nar/gkm899 17962297 PMC2238872

[B14] Dress AWM Flamm C Fritzsch G Grünewald S Kruspe M Prohaska SJ Stadler PF Noisy: Identification of problematic columns in multiple sequence alignments Algorithms for Molecular Biology 2008 3 7 10.1186/1748-7188-3-7 18577231 PMC2464588

[B15] Swingley WD Blankenship RE Raymond J Integrating Markov clustering and molecular phylogenetics to reconstruct the cyanobacterial species tree from conserved protein families Mol Biol Evol 2008 25 643 654 10.1093/molbev/msn034 18296704

[B16] Capella-Gutiérez S Silla-Martínez JM Gabaldón T trimAl: a tool for automated alignment triming in large-scale phylogenetic analyses Bioinformatics 2009 25 1972 1973 10.1093/bioinformatics/btp348 19505945 PMC2712344

[B17] Daubin V Gouy M Perrière G A phylogenomic approach to bacterial phylogeny: evidence of a core of genes sharing a common history Genome Res 2002 12 1080 1090 10.1101/gr.187002 12097345 PMC186629

[B18] Ciccarelli FD Doerks T von Mering C Creevey CJ Snel B Bork P Toward automatic reconstruction of a highly resolved tree of life Science 2006 311 1283 1287 10.1126/science.1123061 16513982

[B19] Adachi J Waddell PJ Martin W Hasegawa M Plastid genome phylogeny and a model of amino acid substitution for protein encoded by chloroplast DNA J Mol Evol 2000 50 348 358 10795826 10.1007/s002399910038

[B20] McMahon MM Sanderson MJ Phylogenetic supermatrix analysis of GenBank sequences from 2228 Papilionoid legumes Syst Biol 2006 55 818 836 10.1080/10635150600999150 17060202

[B21] Schneider TD Stephens RM Sequence logos: a new way to display consensus sequences Nucl Acids Res 1990 18 6097 6100 10.1093/nar/18.20.6097 2172928 PMC332411

[B22] Jayaswal V Jermiin LS Robinson J Estimation of phylogeny using a general Markov model Evol Bioinf Online 2005 1 62 80 PMC2658871 19325854

[B23] Galtier N Gouy M Inferring pattern and process: maximum-likelihood implementation of a nonhomogeneous model of DNA sequence evolution for phylogenetic analysis Mol Biol Evol 1998 15 871 879 9656487 10.1093/oxfordjournals.molbev.a025991

[B24] Jermiin LS Ho SYW Ababneh F Robinson J Larkum AWD The biasing effect of compositional heterogeneity on phylogenetic estimates may be underestimated Syst Biol 2004 53 638 643 10.1080/10635150490468648 15371251

[B25] Phillips MJ Delsuc F Penny D Genome-scale phylogeny and the detection of systematic biases Mol Biol Evol 2004 21 1455 1458 10.1093/molbev/msh137 15084674

[B26] International Union of Pure and Applied Chemistery and International Union of Biochemistery (IUPAC-IUB) Commission on Biochemical Nomenclature Abbreviations and symbols for nucleic acids, polynucleotides and their constituents Biochem J 1970 120 449 454 5499957 10.1042/bj1200449 PMC1179624

[B27] Dayhoff MO Schwartz RM Orcutt BD Dayhoff MO A model of evolutionary change in proteins Atlas of Protein Sequence and Structure 1978 5 Suppl 3 Washington: National Biomedical Research Foundation 345 352

[B28] Embley TM van der Giezen M Horner DS Dyal PL Foster P Mitochondria and hydrogenosomes are two forms of the same fundamental organelle Philos Trans R Soc Lond B Biol Sci 2003 358 191 203 10.1098/rstb.2002.1190 12594927 PMC1693103

[B29] Susko E Roger AJ On reduced amino acid alphabets for phylogenetic inference Mol Biol Evol 2007 24 2139 2150 10.1093/molbev/msm144 17652333

[B30] Stuart A A test for homogeneity of the marginal distributions in a two-way classification Biometrika 1955 42 412 416

[B31] Ababneh F Jermiin LS Ma C Robinson J Matched-pairs tests of homogeneity with applications to homologous nucleotide sequences Bioinformatics 2006 22 1225 1231 10.1093/bioinformatics/btl064 16492684

[B32] International Union of Pure and Applied Chemistery and International Union of Biochemistery (IUPAC-IUB) Commission on Biochemical Nomenclature A one-letter notation for amino acid sequences (definitive rules) Pure Appl Chem 1972 31 639 645 10.1351/pac197231040639

[B33] Von Neumann J Mathematische Grundlagen der Quantenmechanik 1932 Berlin: Springer

[B34] Caffrey DR Somaroo S Hughes JD Mintseris J Huang ES Are protein-protein interfaces more conserved in sequence than the rest of the protein surface? Protein Sci 2004 13 190 202 10.1110/ps.03323604 14691234 PMC2286531

[B35] JAMA: A Java Matrix Package http://math.nist.gov/javanumerics/jama/

[B36] Shannon C A mathematical theory of communication Bell System Tech J 1948 27 379 423 623-656

[B37] Taylor WR The classification of amino acid conservation J Theor Biol 1986 119 205 218 10.1016/S0022-5193(86)80075-3 3461222

[B38] Bordo D Argos P Suggestion for "safe" residue substitutions in site-directed mutagenesis J mol Biol 1991 217 721 729 10.1016/0022-2836(91)90528-E 2005621

[B39] Henikoff S Henikoff JG Amino acid substitution matrices from protein blocks Proc Natl Acad Sci 1992 89 10915 10919 10.1073/pnas.89.22.10915 1438297 PMC50453

[B40] BLOSUM matrices http://blocks.fhcrc.org/blocks/uploads/blosum/

[B41] Eddy SR Where did the BLOSUM62 alignment score matrix come from? Nat Biotechnol 2004 22 1035 1036 10.1038/nbt0804-1035 15286655

[B42] States DJ Gish W Altschul SF Improved sensitivity of nucleic acid database searches using application-specific scoring matrices Methods: A Companion to Methods Enzymol 1991 3 66 70 10.1016/S1046-2023(05)80165-3

[B43] Uesaka H Validity and applicability of several tests for comparing marginal distributions of a square table with ordered categories Behaviormetrika 1991 30 65 78 10.2333/bhmk.18.30_65

[B44] Press WH Teukolsky SA Vetterling WT Flannery BP Numerical Recipes in C -- The Art of Scientific Computing 1992 2 Cambridge: Cambridge University Press

[B45] Artificial datasets used in [48] http://www.atgc-montpellier.fr/phyml/datasets.php

[B46] Rambaut A Grassly NC Seq-Gen: an application for the Monte Carlo simulation of DNA sequence evolution along phylogenetic trees Comput Appl Biosci 1997 13 235 238 9183526 10.1093/bioinformatics/13.3.235

[B47] Jones D Taylor W Thornton J The rapid generation of mutation data matrices from protein sequences Comput Appl Biosci 1992 8 275 282 1633570 10.1093/bioinformatics/8.3.275

[B48] Guindon S Gascuel O A simple, fast, and accurate algorithm to estimate large phylogenies by maximum likelihood Syst Biol 2003 52 696 704 10.1080/10635150390235520 14530136

[B49] Edgar RC MUSCLE: multiple sequence alignment with high accuracy and high throughput Nucleic Acids Res 2004 32 1792 1797 10.1093/nar/gkh340 15034147 PMC390337

[B50] Edgar RC MUSCLE: a multiple sequence alignment method with reduced time and space complexity BMC Bioinformatics 2004 5 113 10.1186/1471-2105-5-113 15318951 PMC517706

[B51] Egan JP Signal detection theory and ROC analysis, series in cognition and perception 1975 New York: Academic Press

[B52] Metz CE Basic principles of ROC analysis Semin Nucl Med 1978 8 283 298 10.1016/S0001-2998(78)80014-2 112681

[B53] Swets J Measuring the accuracy of diagnostic systems Science 1988 240 1285 1293 10.1126/science.3287615 3287615

[B54] Fawcett T An introduction to ROC analysis Pattern Recogn Lett 2006 27 861 874 10.1016/j.patrec.2005.10.010

[B55] McStewart W A note on the power of the sign test Ann Math Stat 1941 12 279 303 10.1214/aoms/1177731710

[B56] Dixon WJ Mood AM The statistical sign test J Am Statist Assoc 1946 41 557 566 10.2307/2280577 20279351

[B57] Hemelrijk J A theorem on the sign test when ties are present Proc Nederl Akad Weten Ser A 1952 55 322

[B58] Gascuel O BIONJ: an improved version of the NJ algorithm based on a simple model of sequence data Mol Biol Evol 1997 14 685 695 9254330 10.1093/oxfordjournals.molbev.a025808

[B59] Goloboff P Farris S Nixon KC TNT (Tree analysis using New Technology) ver. 1.0 2000 Published by the authors, Tucumán, Argentina

[B60] Nixon KC The parsimony ratchet, a new method for rapid parsimony analysis Cladistics 1999 15 407 414 10.1111/j.1096-0031.1999.tb00277.x 34902938

[B61] Estabrook GF McMorris FR Meacham CA Comparison of undirected phylogenetic trees based on subtrees of four evolutionary units Syst Zool 1985 34 193 200 10.2307/2413326

[B62] Felsenstein J Confidence limits on phylogenies: an approach using the bootstrap Evolution 1985 39 783 791 10.2307/2408678 28561359

[B63] Anisimova M Gascuel O Approximate likelihood ratio test for branches: a fast, accurate and powerful alternative Syst Biol 2006 55 539 552 10.1080/10635150600755453 16785212

[B64] Guindon S Dufayard JF Lefort V Anisimova M Hordijk W Gascuel O New algorithms and methods to estimate maximum-likelihood phylogenies: assessing the performance of PhyML 3.0 Syst Biol 2010 59 307 321 10.1093/sysbio/syq010 20525638

[B65] Hanley JA McNeil BJ The meaning and the use of the area under a receiver operating characteristic (ROC) curve Radiology 1982 143 29 36 7063747 10.1148/radiology.143.1.7063747

[B66] Fogarty J Baker RS Hudson SE Michael McCool Case studies in the use of ROC curve analysis for sensor-based estimates in human computer interaction Proceedings of Graphics Interface (GI 2005): 09-11 May 2005; Victoria, British Columbia 2005 University of Waterloo 129 136

[B67] Concatenate: a software to build supermatrices of characters http://www.supertriplets.univ-montp2.fr/PhyloTools.php

[B68] Adachi J Hasegawa M Model of amino acid substitution in proteins encoded by mitochondrial DNA J Mol Evol 1996 42 459 468 10.1007/BF02498640 8642615

[B69] Rodríguez-Ezpeleta N Brinkmann H Burey SC Roure B Burger G Löffelhardt W Bohnert HJ Philippe H Monophyly of Primary Photosynthetic Eukaryotes: Green Plants, Red Algae, and Glaucophytes Curr Biol 2005 15 1325 1330 10.1016/j.cub.2005.06.040 16051178

[B70] Hackett JD Yoon HS Li S Reyes-Prieto A Rümmele SE Bhattacharya D Phylogenomic analysis supports the monophyly of Cryptophytes and Haptophytes and the association of Rhizaria with Chromalveolates Mol Biol Evol 2007 24 1702 1713 10.1093/molbev/msm089 17488740

[B71] Burki F Shalchian-Tabrizi K Pawlowski J Phylogenomics reveals a new 'metagroup' including most photosynthetic eukaryotes Biol Lett 2008 4 366 369 10.1098/rsbl.2008.0224 18522922 PMC2610160

[B72] Hampl V Hug L Leigh JW Dacks JB Lang BF Simpson AGB Roger AJ Phylogenomic analyses support the monophyly of Excavata and resolve relationships among eukaryotic "supergroups" Proc Natl Acad Sci USA 2009 106 3859 3864 10.1073/pnas.0807880106 19237557 PMC2656170

[B73] Ewens W Grant G Statistical methods in bioinformatics: an introduction 2005 Second New York: Springer

[B74] Felsenstein J Evolutionary tree from DNA sequences: a maximum likelihood approach J Mol Evol 1981 17 368 376 10.1007/BF01734359 7288891

[B75] Rzhetsky A Nei M Tests of applicability of several substitution models for DNA sequence data Mol Biol Evol 1995 12 131 151 7877488 10.1093/oxfordjournals.molbev.a040182

[B76] Bowker AH A test for symmetry in contingency tables J Am Stat Assoc 1948 43 572 574 10.2307/2280710 18123073

[B77] Rokas A Williams BL King N Carroll SB Genome-scale approaches to resolving incongruence in molecular phylogenies Nature 2003 425 798 804 10.1038/nature02053 14574403

[B78] Yeast multi-gene dataset http://systbio.org/files/Ren_etal_Rokas2003data.txt

[B79] Lanave C Preparata G Saccone C Serio G A new method for calculating evolutionary substitution rates J Mol Evol 1984 20 86 93 10.1007/BF02101990 6429346

[B80] Rodriguez R Oliver JL Marin A Medina JR The general stochastic model of nucleotide substitution J Theor Biol 1990 142 485 501 10.1016/S0022-5193(05)80104-3 2338834

[B81] Yang Z Estimating the pattern of nucleotide substitution J Mol Evol 1994 39 105 111 8064867 10.1007/BF00178256

[B82] Lake JA Reconstructing evolutionary trees from DNA and protein sequences: paralinear distances Proc Natl Acad Sci USA 1994 91 1455 1459 10.1073/pnas.91.4.1455 8108430 PMC43178

[B83] Lockhart PJ Steel MA Hendy MD Penny D Recovering evolutionary trees under a more realistic model of sequence evolution Mol Biol Evol 1994 11 605 612 19391266 10.1093/oxfordjournals.molbev.a040136

[B84] Steel MA Recovering a tree from the leaf colourations it generates under a Markov model Appl Math Lett 1994 7 19 23 10.1016/0893-9659(94)90024-8

[B85] Taylor DJ Piel WH An assessment of accuracy, error, and conflict with support values from genome-scale phylogenetic data Mol Biol Evol 2004 21 1534 1537 10.1093/molbev/msh156 15140947

[B86] Ren F Tanaka H Yang Z An empirical examination of the utility of codon-substitution models in phylogeny reconstruction Syst Biol 2005 54 808 818 10.1080/10635150500354688 16243764

[B87] Burleigh JG Driskell AC Sanderson MJ Supertree bootstrapping methods for assessing phylogenetic variation among genes in genome-scale data sets Syst Biol 2006 55 426 440 10.1080/10635150500541722 16861207

[B88] Ané C Larget B Baum DA Smith SD Rokas A Bayesian estimation of concordance among gene trees Mol Biol Evol 2007 24 412 426 10.1093/molbev/msl170 17095535

[B89] Criscuolo A Michel CJ Phylogenetic inference with weighted codon evolutionary distances J Mol Evol 2009 68 377 392 10.1007/s00239-009-9212-y 19308635

[B90] Néron B Ménager H Maufrais C Joly N Maupetit J Letort S Carrere S Tuffery P Letondal C Mobyle: a new full web bioinformatics framework Bioinformatics 2009 25 3005 3011 10.1093/bioinformatics/btp493 19689959 PMC2773253

